# EfficientUNetViT: Efficient Breast Tumor Segmentation Utilizing UNet Architecture and Pretrained Vision Transformer

**DOI:** 10.3390/bioengineering11090945

**Published:** 2024-09-21

**Authors:** Shokofeh Anari, Gabriel Gomes de Oliveira, Ramin Ranjbarzadeh, Angela Maria Alves, Gabriel Caumo Vaz, Malika Bendechache

**Affiliations:** 1 Department of Accounting, Economic and Financial Sciences, Islamic Azad University, South Tehran Branch, Tehran 1584743311, Iran; shokofehanarii@gmail.com; 2 Poli.TIC—CTI—Renato Archer, Campinas 13069-901, Brazil; amalves@cti.gov.br; 3 School of Computing, Faculty of Engineering and Computing, Dublin City University, D09 V209 Dublin, Ireland; ramin.ranjbarzadehkondrood2@mail.dcu.ie; 4 School of Electrical and Computer Engineering, State University of Campinas, Campinas 13083-852, Brazil; g226919@dac.unicamp.br; 5 ADAPT Research Centre, School of Computer Science, University of Galway, H91 TK33 Galway, Ireland; malika.bendechache@universityofgalway.ie

**Keywords:** breast cancer, UNet, vision transformer, depthwise separable convolutional

## Abstract

This study introduces a sophisticated neural network structure for segmenting breast tumors. It achieves this by combining a pretrained Vision Transformer (ViT) model with a UNet framework. The UNet architecture, commonly employed for biomedical image segmentation, is further enhanced with depthwise separable convolutional blocks to decrease computational complexity and parameter count, resulting in better efficiency and less overfitting. The ViT, renowned for its robust feature extraction capabilities utilizing self-attention processes, efficiently captures the overall context within images, surpassing the performance of conventional convolutional networks. By using a pretrained ViT as the encoder in our UNet model, we take advantage of its extensive feature representations acquired from extensive datasets, resulting in a major enhancement in the model’s ability to generalize and train efficiently. The suggested model has exceptional performance in segmenting breast cancers from medical images, highlighting the advantages of integrating transformer-based encoders with efficient UNet topologies. This hybrid methodology emphasizes the capabilities of transformers in the field of medical image processing and establishes a new standard for accuracy and efficiency in activities related to tumor segmentation.

## 1. Introduction

Breast cancer continues to be a highly common and lethal type of cancer that affects women globally [1,2]. A mass of aberrant cells that develops within the breast tissue is called a breast tumor, which can be benign or malignant. Because they can spread (metastasize) to other parts of the body and cause major health issues as well as, frequently, death, malignant tumors are especially dangerous [3]. Effective treatment and higher survival rates depend on early discovery and precise diagnosis. The heterogeneity in tumor size, form, and appearance on medical imaging, however, presents substantial barriers to the process of identifying and recognizing breast cancers [4,5]. 

Many imaging modalities, such as mammography, ultrasound, and magnetic resonance imaging (MRI), can identify and detect breast cancers. The most popular screening method is mammography, which produces finely detailed images of the breast using low-dose X-rays. Using high-frequency sound waves to create images, ultrasound is especially helpful in differentiating between cysts filled with fluid and solid tumors. Conversely, magnetic resonance imaging (MRI) yields high-contrast images and is frequently employed for high-risk patients or to delve deeper into unclear results from other imaging modalities. Although each technique has advantages and disadvantages, correctly interpreting the images they generate is essential to a successful diagnosis [1,6]. 

One of the most important steps in the diagnostic workflow is the segmentation of breast tumors, which is the act of defining the boundaries of the tumor inside an image. This has always been performed manually by radiologists, which takes a lot of time and is prone to differences in observation quality [7,8,9]. The process of manual segmentation necessitates a high degree of expertise and can be error-prone because it includes delineating the tumor on each image slice. Computational algorithm-driven automated segmentation techniques seek to address these issues by delivering repeatable and consistent outcomes [10,11,12]. 

Automated breast tumor segmentation, however, provides numerous benefits. It offers reliable and replicable outcomes, lessens the burden on healthcare practitioners, and enables quick analysis of extensive information. In addition, automated techniques can identify intricate patterns and characteristics that may go unnoticed by human observation, hence potentially resulting in timelier and more precise diagnoses [13,14]. Conventional image processing methods frequently encounter difficulties when dealing with the diversity and intricacy of medical images. Deep learning (DL) approaches have become effective tools for analyzing medical images in order to tackle these difficulties [7,15].

Convolutional neural networks (CNNs), in particular, are DL models that have shown a remarkable ability to learn intricate patterns and features from vast datasets [4,16,17]. DL models have the ability to automatically extract hierarchical features from raw images, in contrast to typical machine learning (ML) techniques that necessitate manual feature extraction. Because of this flexibility, they are especially well suited for the complex task of segmenting breast tumors, where there can be a significant degree of diversity in tumor appearance [15,18,19,20,21]. 

The consistency and scalability of the outcomes are two main ways that human and automatic segmentations differ from one another. Because of the radiologist’s experience, manual segmentation may be more accurate in specific circumstances, but it is impractical for handling massive amounts of data. On the other hand, automated segmentation with DL methods can process large volumes of imaging data quickly and reliably, lessening the effort required of medical personnel and lowering the possibility of human error. Furthermore, when more annotated data become available, DL models can adjust to new imaging modalities and changing clinical practices, enhancing their performance over time [17,22,23,24]. 

There are numerous advantages to using DL for breast tumor segmentation. These models help with early and accurate tumor border delineation, which is crucial for effective treatment outcomes. They also improve the precision of tumor boundary delineation. Moreover, multi-modal imaging data can be included in DL models, offering a thorough analysis that incorporates the advantages of several imaging modalities [23,25]. Personalized treatment regimens and better clinical decision making may result from this all-encompassing strategy. Deep learning’s potential to revolutionize breast cancer diagnosis and treatment could be realized as the field develops, eventually leading to better patient outcomes and survival rates [22,25].

Chen et al. [26] have recently tackled the difficulties presented by the intricate ultrasound patterns and the varying shapes and sizes of tumors in the segmentation of breast tumors. An advanced selective kernel convolution was developed for the segmentation of breast tumors. This technique incorporates several representations of feature map sections and dynamically adjusts the weights of these areas based on both spatial and channel dimensions. The implementation of this region calibration approach permits the network to concentrate more efficiently on region features that have a significant impact while minimizing the influence of less valuable regions. To increase the accuracy of segmentation, they integrate the enhanced selective kernel convolution into a U-net architecture with deep supervision restrictions. This allows for the adaptive capture of strong representations of breast cancers. By tackling the complexities of breast tumor segmentation from ultrasound images, this method shows a considerable improvement in terms of robustness and accuracy.

Tagnamas et al. [4] introduced an innovative approach for segmenting and classifying breast tumors. They utilized a two-encoder architecture that integrates EfficientNetV2 with a modified ViT encoder. The EfficientNetV2 backbone is employed to maintain local information in breast ultrasound (BUS) images, while the transformer encoder utilizes a self-attention (SA) method to capture a broad spectrum of high-level and intricate features. To efficiently combine the features obtained from both encoders, the authors devised a Channel Attention Fusion (CAF) module. This module selectively enhances significant features, hence enhancing the integration of local and high-level information. These feature maps that have been combined are subsequently reconstructed employing a decoder to create segmentation maps. In addition, a straightforward and efficient classifier based on the MLP-Mixer is used to categorize the segmented tumor regions into benign and malignant categories. This classifier, utilized for the initial time in lesion classification in BUS images, showcases a groundbreaking and efficient method for breast tumor segmentation and classification.

Zhu et al. [14] propose a new method for segmenting breast tumors by utilizing a Swin-Net architecture that combines CNNs and transformers. This integration improves the accuracy of breast ultrasound image segmentation. The technique employs the swing-transformer model due to its robust global modeling capability and accurate feature extraction capabilities. The Swin-Net framework has two novel modules: the feature refinement and improvement module (RLM) and the hierarchical multi-scale feature fusion module (HFM). The RLM module improves and strengthens the characteristics acquired by the transformer encoder, while the HFM module handles multi-scale low-level details and high-level semantic characteristics to provide efficient integration of features across layers, reduction in noise, and enhanced segmentation performance. 

To optimize the effectiveness and precision of tumor segmentation and overcome the problems of varying shapes and sizes of tumors, this work presents the EfficientUNetViT model, which is a hybrid architecture that combines the advantages of U-Net, depthwise separable convolutions, and transformer. 

The paper’s structure is arranged as follows: We go over each of our approach’s components in Section 2. The UNet architecture with depthwise separable convolutions and ViT is described in Section 2.1. The datasets utilized for training and evaluation, together with preprocessing techniques, are described in Section 2.2. The augmentation approaches used to improve dataset variety are covered in Section 2.3. The training setup, settings, and optimization algorithms are described in depth in Section 2.4. In Section 3, the metrics used to evaluate the model’s performance are defined in Section 3.1. Experimental results, comparisons with other approaches, and a discussion of the results are presented in Section 3.2. The main contributions, clinical practice implications, and future research prospects are outlined in Section 4.

## 2. Materials and Methods

### 2.1. Model Structure

In this work, we used a pretrained ViT model with a UNet architecture to perform breast tumor segmentation. The UNet architecture is a widely recognized neural network specifically developed for the purpose of segmenting biological images. The system comprises a contracting pathway for context acquisition and a symmetric expanding pathway for reliable localization. The contracting route follows the conventional structure of a convolutional network, wherein each stage comprises two 3 × 3 convolutions, succeeded by a rectified linear unit (ReLU) and a 2 × 2 max pooling operation. Conversely, the expanding approach involves increasing the resolution of the feature map by upsampling and then using a 2 × 2 convolution (up-convolution) to reduce the number of feature channels by half. This is followed by concatenating the resulting feature map with the equivalent cropped feature map from the contracting path.

Our UNet utilizes depthwise separable convolutional blocks, which are a type of convolution developed to decrease the computational cost and parameters of the model while maintaining performance. Depthwise separable convolutions divide the convolution operation into two distinct layers: Depthwise convolutions, which utilize one filter for each input channel, and pointwise convolutions, which use a 1 × 1 convolution to merge the outputs of the depthwise layer [27,28]. This method greatly decreases the quantity of parameters and computing burden, resulting in a more efficient and expedited training process for the network, while yet retaining a high level of accuracy [27,29].

The UNet’s depthwise separable convolutional blocks offer numerous benefits. Firstly, they aid in mitigating overfitting by reducing the number of parameters, which is particularly important when working with tiny datasets that are frequently seen in medical imaging. Additionally, the decreased computational complexity enables the utilization of more complicated networks or larger batches, thereby enhancing the model’s performance even further. Finally, these blocks maintain the spatial hierarchies and characteristics acquired at various layers, guaranteeing that the crucial details for segmentation tasks are conserved across the network [27,30].

The main objective of adding a ViT to the UNet architecture is to make use of the transformers’ strong feature extraction capabilities, which have lately demonstrated exceptional performance on a range of computer vision applications [31,32]. Vision transformers provide a major improvement over conventional CNNs by using self-attention processes to capture global context and dependencies inside an image. ViTs are a strong substitute for CNNs in image analysis due to their capacity to represent long-range interactions and contextual linkages [33,34]. 

By integrating ViT into the UNet architecture, the model may take advantage of the advantages of both worlds: the global context knowledge offered by ViT and the accurate localization and segmentation capabilities of UNet. This combination improves the model’s ability to capture both local and global information, which improves the model’s ability to appropriately segment breast cancers [32,35].

The ViT functions by partitioning an image into a series of patches, seeing each patch as a token similar to words in natural language processing. The series of patches is subsequently fed into a transformer model, which utilizes self-attention to record the interconnections among the patches. By employing this method, ViT is able to effectively capture extensive connections and contextual details throughout the entire image, surpassing the capabilities of conventional CNNs that typically depend on limited receptive fields. ViT has demonstrated exceptional performance in image classification challenges by capturing global context. This success has inspired us to apply ViT to segmentation tasks in our study [31,35,36].

Our UNet architecture utilized a pretrained ViT model as the encoder. The choice to using a pretrained model was motivated by numerous benefits. Pretrained models have acquired extensive feature representations from vast datasets, allowing them to exhibit superior generalization capabilities when faced with novel problems and limited data. This transfer learning strategy greatly diminishes the requirement for abundant labeled data, which is especially advantageous in the field of medical imaging where annotated datasets are frequently limited. In addition, pretrained models accelerate the training process by leveraging existing information instead of starting from the beginning.

**Figure 1:**
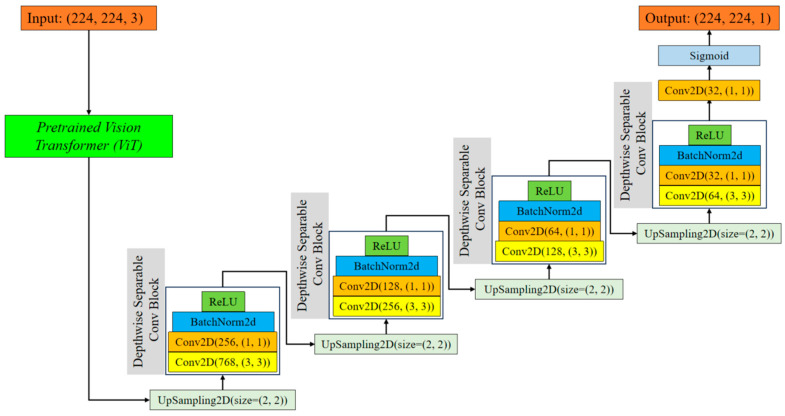
Structure of the proposed EfficientUNetViT model.

Integrating a pretrained ViT into our UNet model has several advantages. The ViT encoder captures salient information from the input images, yielding a resilient representation for the succeeding decoder stages. These characteristics collect both specific and overall information, improving the model’s capacity to accurately define tumor boundaries. The ViT encoder utilizes the self-attention mechanism to identify important areas in the image that are crucial for accurate segmentation. This enhances the overall performance of the model [35,36]. Figure 1 indicates structure of the proposed EfficientUNetViT model.

### 2.2. Dataset

This study utilizes the Breast Ultrasound Image (BUSI) dataset to assess the effectiveness of our breast tumor segmentation model [37]. The BUSI dataset, gathered in 2018, consists of 780 ultrasound images obtained from 600 female patients between the ages of 25 and 75. The images are classified into three categories: malignant, benign, and normal. The images initially exist in PNG format and exhibit a diverse range of dimensions, contours, and tumor characteristics, which presents considerable difficulty for automatic segmentation approaches. An image typically has dimensions of 500 pixels by 500 pixels. To ensure consistency in the input for the model, all images in the dataset were scaled to a uniform dimension of 224 × 224 pixels, with three color channels (224, 224, 3). The act of resizing guarantees uniformity and achieves a harmonious balance between computational effectiveness and the retention of adequate data for precise segmentation. In addition, the pixel values are rescaled to a range of 0 to 1 to expedite the convergence process during model training. 

The BUSI dataset contains meticulous annotations for each image, accurately outlining the precise boundaries of the tumors. The annotations are presented as binary masks, with tumor locations indicated by a pixel value of 1 and background pixels represented by a value of 0. These masks function as the ultimate point of reference for both training and assessing the segmentation model. To guarantee an equitable evaluation of the model’s effectiveness, the dataset is partitioned into three distinct subsets: training, validation, and test sets. The training set consists of 80% of the images; the validation set contains 10% of the images, and the test set includes the remaining 10% of the images. This division guarantees that the model is trained on a varied assortment of images and evaluated on unfamiliar data to assess its ability to generalize.

### 2.3. Data Augmentation

This study utilizes a comprehensive data augmentation technique to enhance the resilience and generalization capabilities of our EfficientUNetViT model. Data augmentation is an essential method in DL, especially when working with restricted datasets [38,39]. The augmentation approach used involves a proprietary transform function that guarantees that the image and its matching mask undergo the same transformations, ensuring spatial consistency between them. In addition to random rotations up to 20 degrees, the main data augmentation pipeline also contains random flips, both vertical and horizontal. These augmentations are designed to improve the model’s resilience by exposing it to diverse viewpoints of the tumor images. The transformations are implemented by composing a series of operations that convert images to the PIL format for modification and then convert them back to tensors. In addition, a distinct transformation pipeline is established for the validation and testing steps. This pipeline solely focuses on converting the data into tensor format, guaranteeing that the images and masks remain unaltered during these crucial assessment stages. These adjustments replicate the diversity observed in actual clinical situations, aiding the model in becoming immune to alterations in tumor location, dimensions, and alignment [40,41].

### 2.4. Training Procedure

The training approach of our EfficientUNetViT model is specifically intended to achieve optimal performance and enhance generalization capabilities. At first, the dataset is partitioned into training, validation, and test sets. Data augmentation is applied to the training set to artificially increase the dimension of the dataset and create variability. This technique is used to prevent overfitting. 

There is an early termination criterion if the validation loss does not show progress after 10 iterations in the 50 iterations of the training method. When the model has adequately learned the underlying patterns in the data, the training process is stopped, preventing overfitting to the irrelevant details. The model keeps its capacity to generalize on unknown data by doing this. To balance the requirement for adequate learning progress without significantly affecting the model’s performance, the learning rate (LR) is set at 1 × 10^−4^. 

The training procedure uses the Adam optimizer, which is renowned for its effectiveness and flexible learning rate capabilities. Adam excels at handling noisy situations and sparse gradients because it incorporates the best features of AdaGrad and RMSProp, two additional extensions of stochastic gradient descent. Its adjustable LR facilitates more rapid and stable convergence by dynamically modifying the learning rate during training. In order to minimize the risk of overfitting and maintain the model’s performance and reliability, the combination of an early stopping, robust optimizer, and a controlled LR helps the model learn from the data effectively [42,43].

Throughout each epoch, the model undergoes training using batches of augmented training data. The model’s parameters are changed for each batch via backpropagation to minimize the binary cross-entropy loss between the true and predicted segmentation masks. Following every epoch, the model’s performance is evaluated on the validation set. The validation loss and metrics are utilized to ascertain if early stopping should be initiated and to ascertain the optimal model based on validation performance. The optimal model parameters are stored to guarantee the utilization of the most efficient model for testing. 

Ultimately, the optimal model is loaded and its performance is assessed on the test set to determine its ability to generalize. This systematic training method guarantees that the model not only achieves high performance on the training data but also exhibits effective generalization to novel, unseen data.

## 3. Experiments

### 3.1. Evaluation Metrics

The study assessed our breast tumor segmentation models’ performance employing a range of established measures to ensure a thorough evaluation of their efficacy and accuracy. Intersection over union (IoU), recall, precision, and dice similarity coefficient (Dice) were the main metrics that were employed. Precision gauges how well positive predictions come true, including important information about how well the model reduces false positives. To prevent the model from mistakenly classifying non-tumorous areas as tumors, this metric is crucial. Recall, on the other hand, indicates how well the model detects accurate tumor locations by measuring its accuracy in identifying all pertinent occurrences [1,26,44,45]. 

The overlap between the anticipated segmentation and the ground truth is measured by the IoU metric. IoU is a trustworthy indicator of spatial prediction accuracy since it shows how well the model’s segmentations match the real tumor regions. Similarly, when assessing the overlap between anticipated and true segmentations, the dice coefficient highlights the harmonic average of accuracy and recall, providing a fair assessment of both measures. Because it takes into account both the accuracy and completeness of the segmentations, the dice coefficient is very useful for evaluating the quality of segmentation outputs. By using these criteria, we make sure that the performance of our model in breast tumor segmentation is thoroughly and nuancedly evaluated, offering important insights into its therapeutic value [8,25,46,47].

### 3.2. Results and Discussion

**Figure 2:**
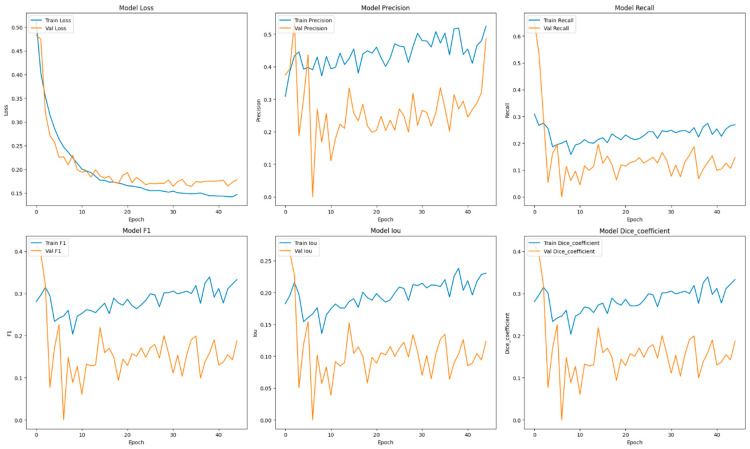
Training dynamics over epochs, uncovering the progression of loss, recall, and precision for the EfficientUNetViT model (ViT + UNet + Augmentation) utilized in breast tumor segmentation.

**Figure 3:**
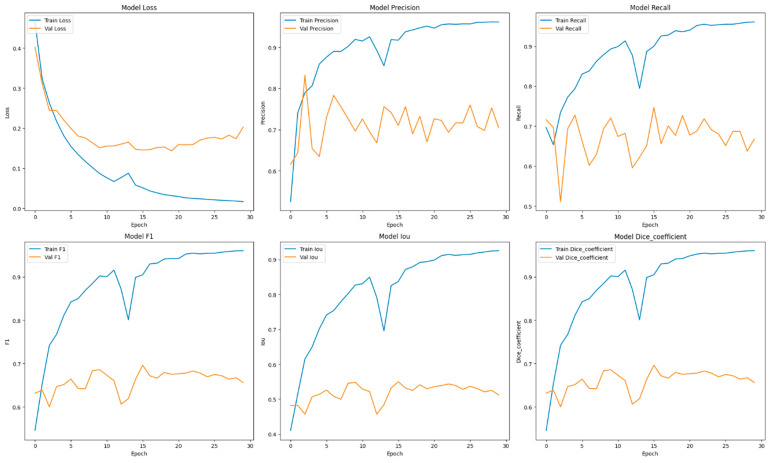
Training dynamics over epochs, uncovering the progression of loss, recall, and precision for the EfficientUNetViT model (ViT + UNet) utilized in breast tumor segmentation.

**Figure 4:**
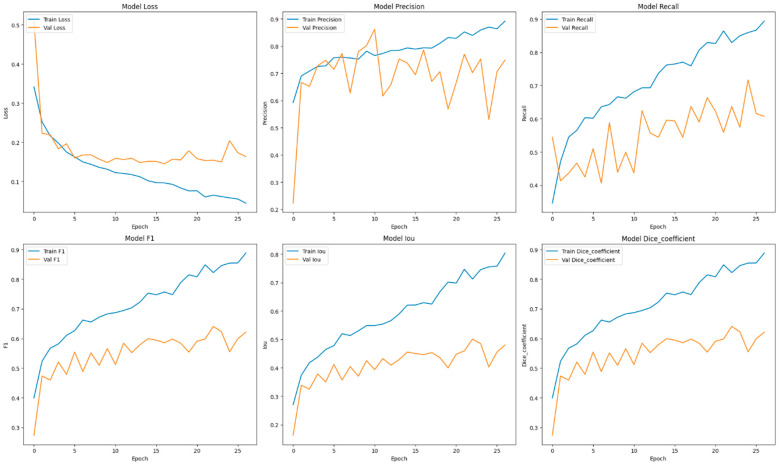
Training dynamics over epochs, uncovering the progression of loss, recall, and precision for the UNet model utilized in breast tumor segmentation.

**Figure 5:**
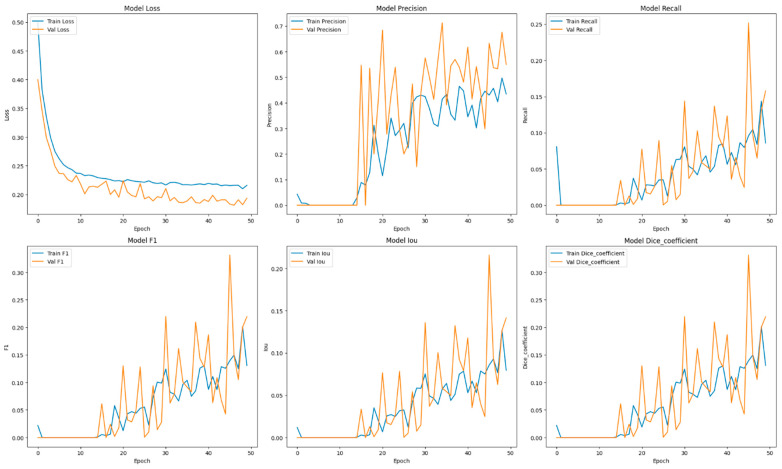
Training dynamics over epochs, uncovering the progression of loss, recall, and precision for the UNet + Augmentation model utilized in breast tumor segmentation.

**Figure 6:**
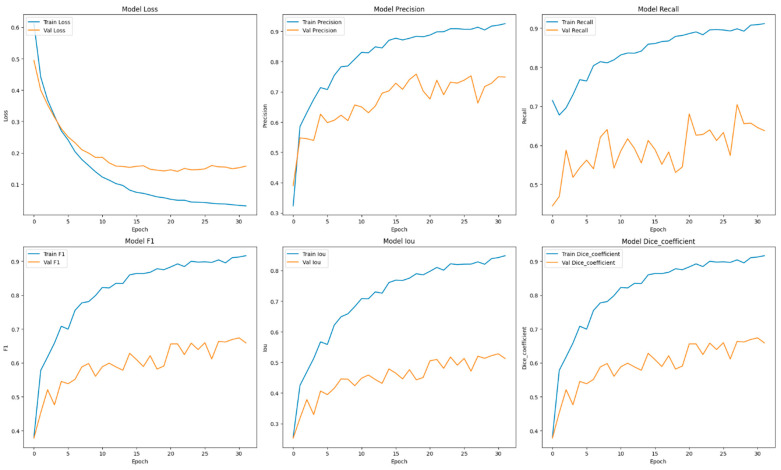
Training dynamics over epochs, uncovering the progression of loss, recall, and precision for the EfficientNet + UNet model utilized in breast tumor segmentation.

**Figure 7:**
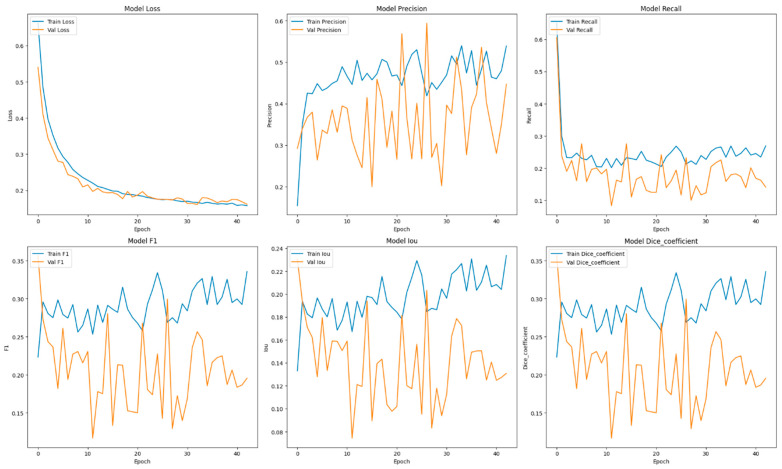
Training dynamics over epochs, uncovering the progression of loss, recall, and precision for the EfficientNet + UNet + Augmentation model utilized in breast tumor segmentation.

This section focuses on the presentation and analysis of the performance of the EfficientUNetViT model in segmenting breast tumors. The effectiveness of the model is assessed by measuring many important metrics, such as IoU, precision, F1 score, recall, and dice coefficient, on both the validation and test datasets. In addition, we analyze the influence of data augmentation and the incorporation of the ViT and EfficientNet into the UNet architecture on the accuracy of segmentation. The results indicate that our method greatly improves the accuracy of segmenting breast tumors, providing a reliable and efficient solution for automated segmentation. Table 1 presents comprehensive comparisons of the training time effectiveness between different models. The training dynamics over epochs of models are indicated in Figure 2, Figure 3, Figure 4, Figure 5, Figure 6 and Figure 7.

The UNet model is a renowned CNN architecture specifically tailored for the purpose of biomedical image segmentation applications. Within the BUSI dataset, UNet exhibited robust performance, achieving a precision of 0.8281 and a recall of 0.6122. These results indicate UNet’s effectiveness in accurately segmenting breast tumor images. The F1 score and dice coefficient achieved a value of 0.6622, indicating a well-balanced performance in terms of precision and recall. The IoU of 0.5311 highlights the ability of the model to produce precise segmentation masks, while there is still potential for improvement in accurately capturing the entire tumor boundaries.

The integration of UNet with data augmentation approaches resulted in a substantial decrease in the model’s performance across all metrics. The precision decreased to 0.5123, while the recall experienced a significant decline to 0.1420, leading to a notably lower F1 score of 0.2087 and an IoU of 0.1372. The dice coefficient fell to 0.2087. These findings indicate that the augmentation strategies used may not have been appropriate for the UNet model, which could have resulted in overfitting or misalignment between the images and masks during training, thus diminishing the model’s efficacy.

The integration of EfficientNet as the underlying framework with a UNet architecture seeks to capitalize on EfficientNet’s robust feature extraction capabilities alongside UNet’s segmentation prowess. The model attained a precision of 0.7691 and a recall of 0.6638, resulting in an F1 score of 0.6916 and a dice coefficient that matches the F1 score. The IoU value of 0.5514 suggests that this model outperformed the standalone UNet in terms of accurately recognizing and segmenting the tumor locations. In general, this combination resulted in a significant enhancement in performance, namely in terms of recall, indicating its improved capability to accurately detect tumor locations.

By incorporating data augmentation, the performance of the EfficientNet + UNet model exhibited a decline in comparison to its non-augmented version. The precision decreased to 0.5978, while the recall and F1 scores were 0.3141 and 0.3869, respectively. The IoU and dice coefficient both yielded values of 0.2760 and 0.3869, respectively. These results indicate that although the model benefits from the powerful feature extraction capabilities of EfficientNet, the augmentation approaches employed did not improve and potentially impaired the model’s capacity to generalize effectively on the BUSI dataset.

The model that performed the best in this investigation was the ViT + UNet model, which combines a ViT with the UNet architecture. The model obtained a precision of 0.7901 and a recall of 0.7882, with an F1 score and dice coefficient both equal to 0.7584. The tested models had an IoU of 0.6292, which was the highest, suggesting good segmentation ability. The ViT’s capacity to capture extensive interdependencies within the image data, coupled with UNet’s efficient segmentation, enabled this model to outperform in the identification and demarcation of tumor zones, rendering it a potent tool for breast tumor segmentation.

The ViT + UNet model’s performance significantly decreased when augmentation was added. The precision decreased to 0.5200, while the recall fell to 0.2995. As a result, the F1 score and dice coefficient both stood at 0.3527. The IoU value was 0.2510, indicating that the model’s capacity to accurately segment tumor locations decreased. These findings indicate that, like the other models, the augmentation techniques employed may have produced unwanted noise or distortions that negatively impacted the model’s performance, instead of improving its ability to generalize.

An in-depth examination of the training measures during the epochs offers valuable understanding into the model’s acquisition process. The training of the ViT + UNet model on the BUSI dataset showcases a comprehensive progression of the model’s learning process throughout the epochs (See Figure 2). In the initial phases, particularly in the first few epochs, the model demonstrates fast enhancements in performance indicators, indicating successful acquisition of knowledge from the data. During the transition from Epoch 1 to Epoch 2, there was a notable decrease in the training loss, which went from 0.4652 to 0.3229. This improvement was accompanied by enhancements in precision, recall, and F1 scores. This suggests that the model rapidly adjusted to the characteristics of the breast tumor images. 

Throughout the training process, the model’s performance was consistently and gradually enhanced. At Epoch 6, the model attained a train precision of 0.8761 and a train recall of 0.8301. The validation precision and recall also exhibited favorable patterns, suggesting that the model was efficiently learning without experiencing overfitting at this point. The F1 score and dice coefficient exhibited a strong correlation between the training and validation phases, indicating a consistent enhancement in the model’s capacity to accurately predict both true positives and effectively reduce false negatives. 

During Epochs 7 to 17, the model’s training and validation losses consistently decreased, albeit the pace of the drop was slower, indicating that the model was converging. Throughout this time frame, the F1 scores and dice coefficients consistently demonstrated high values, showing that the model’s accuracy in segmenting remained robust. However, a little deviation between the metrics used for training and validation began to emerge, namely with precision and recall. This indicates that although the model’s confidence in its predictions was growing (as evidenced by the rising precision), there were difficulties in consistently capturing all pertinent characteristics (as shown by occasional changes in recall). 

Subsequent periods, specifically Epoch 20 and onwards, exhibited a more consistent performance, with slight variations in the validation loss and segmentation parameters. By Epoch 20, the precision of the train was 0.9513, but the validation precision was slightly lower at 0.6706. The recall and F1 scores showed similar patterns. The variations found in the model’s performance metrics, namely in the IoU and dice coefficients, may suggest that the model is making efforts to improve its capacity to generalize across the validation set, resulting in the observed stability. 

In the end, the training process was stopped prematurely around Epoch 30 because of early ending criteria, which probably prevented overfitting. Currently, the model attained a high level of precision and recall in both the training and validation stages. The validation F1 scores and dice coefficients fell within the range of 0.6561 to 0.6857, indicating strong performance. Additionally, the IoU values suggest excellent segmentation capabilities. Overall, the ViT + UNet model exhibited a strong training process, resulting in a highly generalized model for breast tumor segmentation. However, it was crucial to carefully manage accuracy and recall to prevent potential overfitting as the model progressed.

The learning curves demonstrated that the models without augmentation consistently and significantly improved in performance, whereas the model with augmentation exhibited notable instability. The instability observed suggests that the augmentation strategies used may not have been well suited for the dataset, perhaps resulting in a decrease in the model’s capacity to generalize from the enhanced data. 

**Figure 8:**
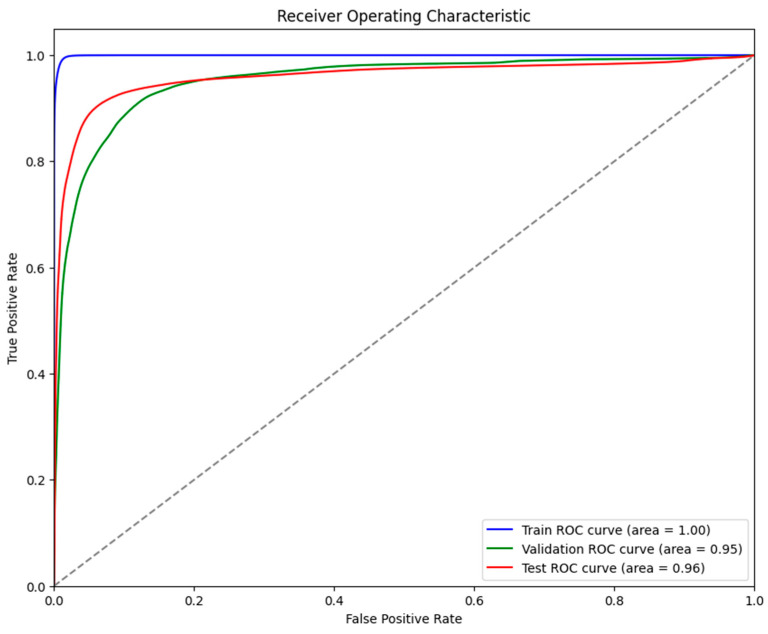
Displaying the ROC of the ViT + UNet model.

**Figure 9:**
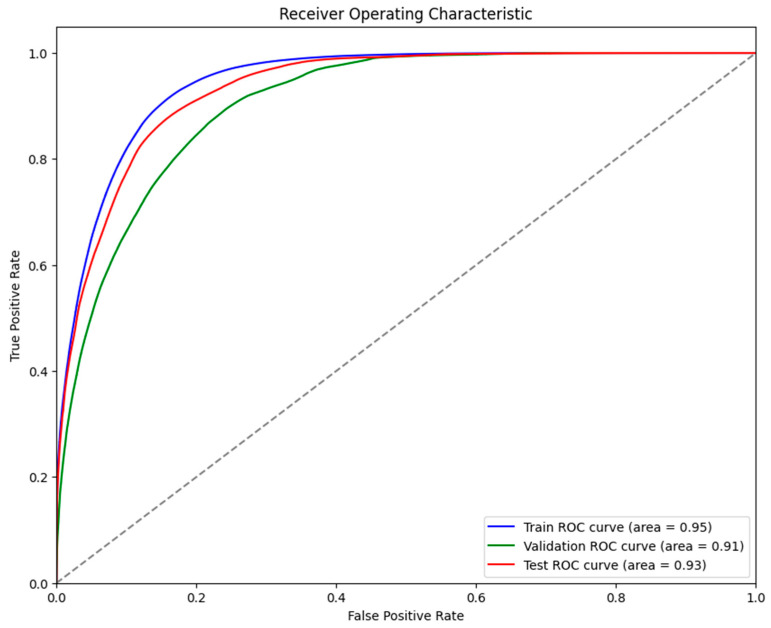
Displaying the ROC of the ViT + UNet + Augmentation model.

**Figure 10:**
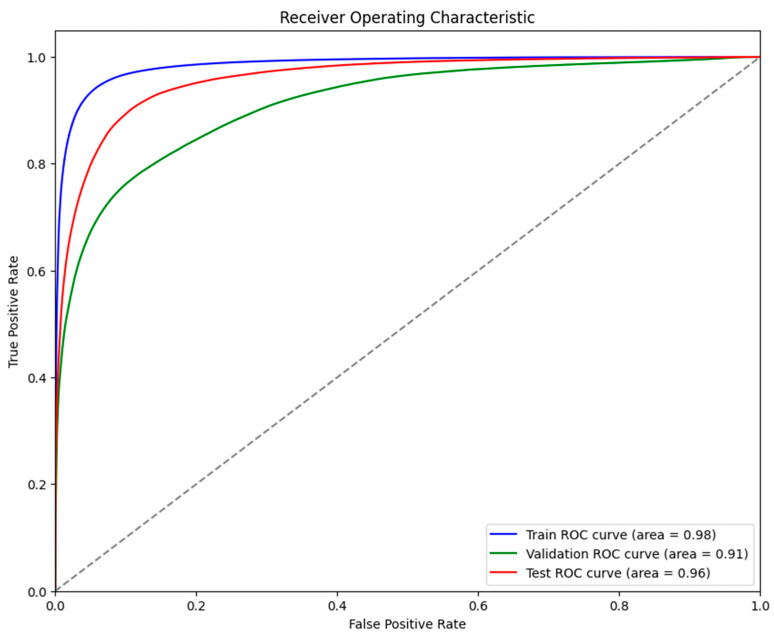
Displaying the ROC of the UNet model.

**Figure 11:**
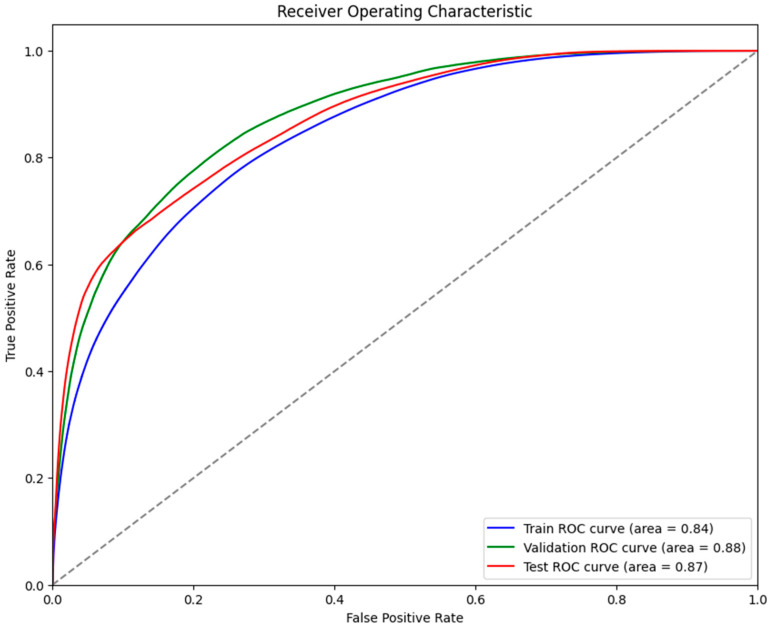
Displaying the ROC of the UNet + Augmentation model.

**Figure 12:**
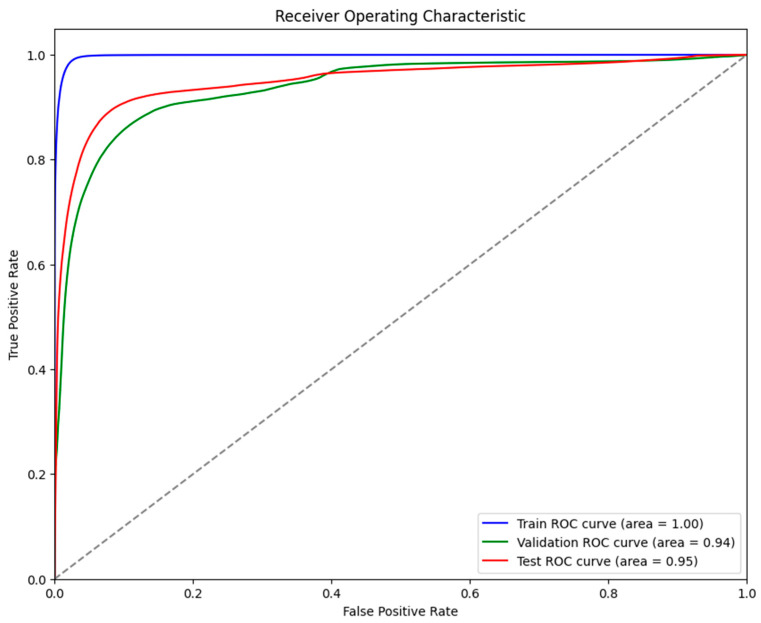
Displaying the ROC of the EfficientNet + UNet model.

**Figure 13:**
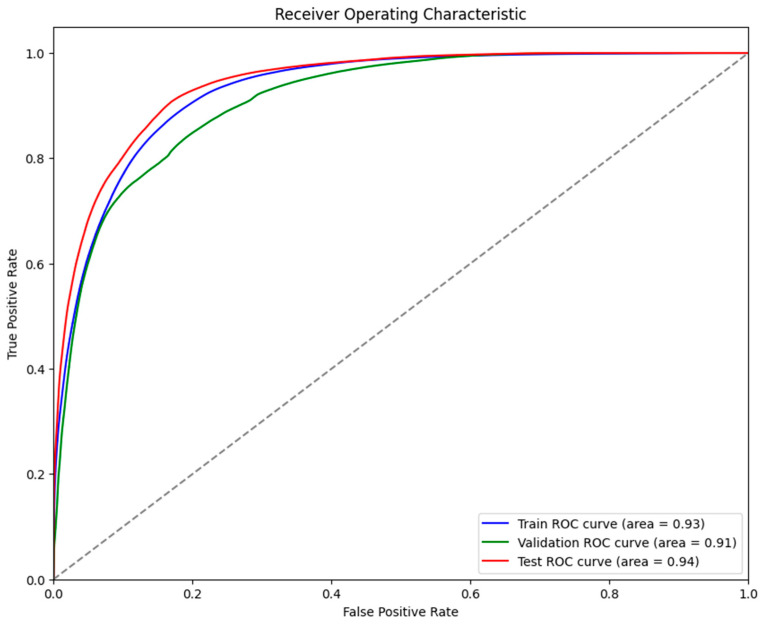
Displaying the ROC of the EfficientNet + UNet + Augmentation model.

It was evident from the learning curves that the performance trajectories of the augmented and non-augmented models differed significantly. The augmented model showed notable instability, whereas the non-augmented model showed a consistent and significant gain in performance with time. This instability raises the possibility that the augmentation methods used were not ideal for the particular features of the dataset. As a result, the enhanced model encountered difficulties while attempting to make generalizations from the enhanced data, which could have limited its capacity to acquire strong characteristics that are essential for accurate segmentation. The steady growth of the non-augmented model emphasizes how important it is to use augmentation procedures that are appropriate and customized to the particular properties of the dataset. The ROC of the different models are indicated in Figure 8, Figure 9, Figure 10, Figure 11, Figure 12 and Figure 13.

**Figure 14:**
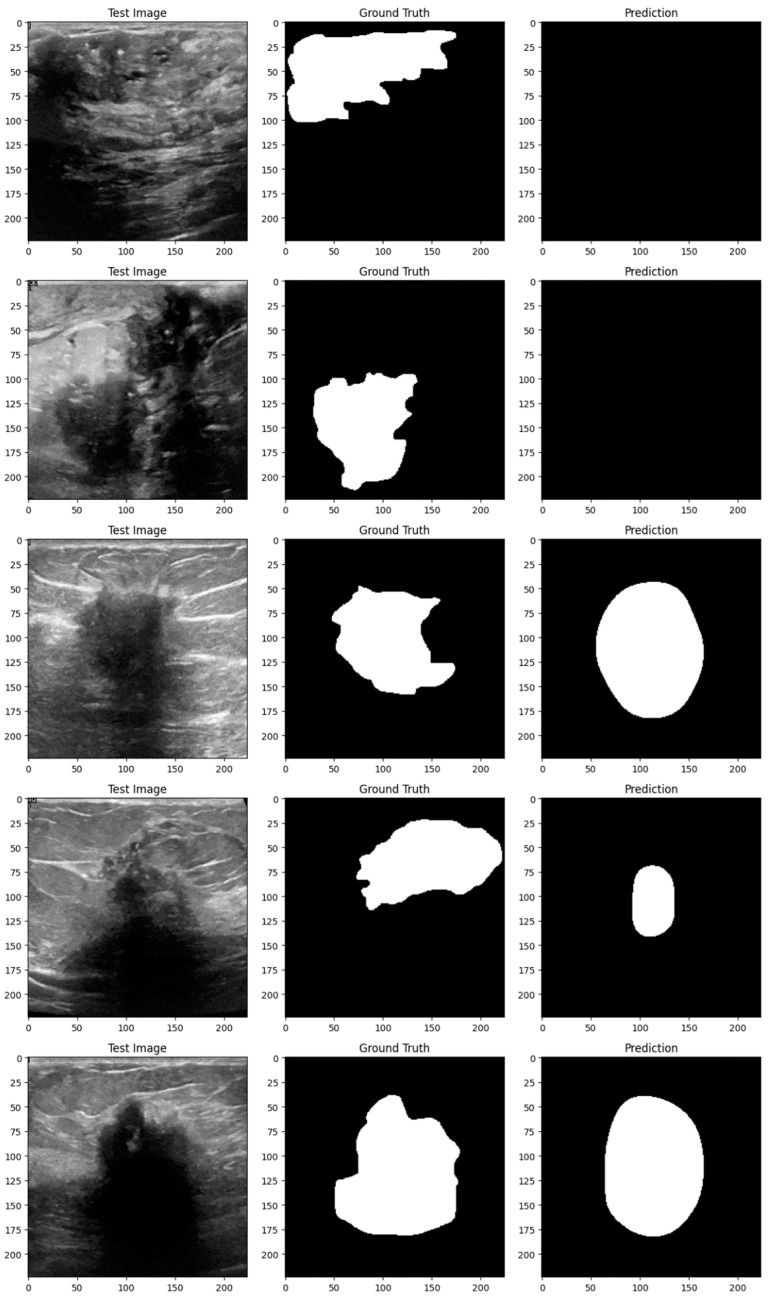
Displaying the model’s predictions on test samples (ViT + UNet + Augmentation).

**Figure 15:**
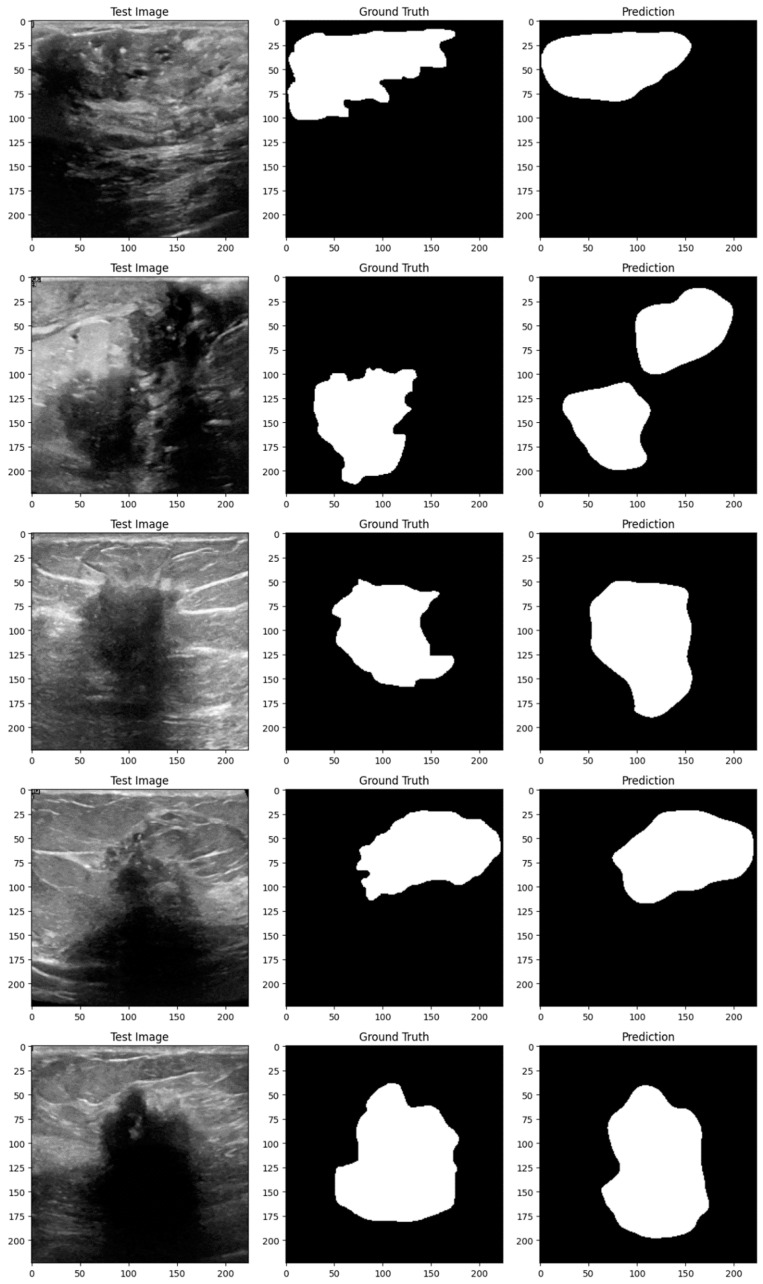
Displaying the model’s predictions on test samples (ViT + UNet).

**Figure 16:**
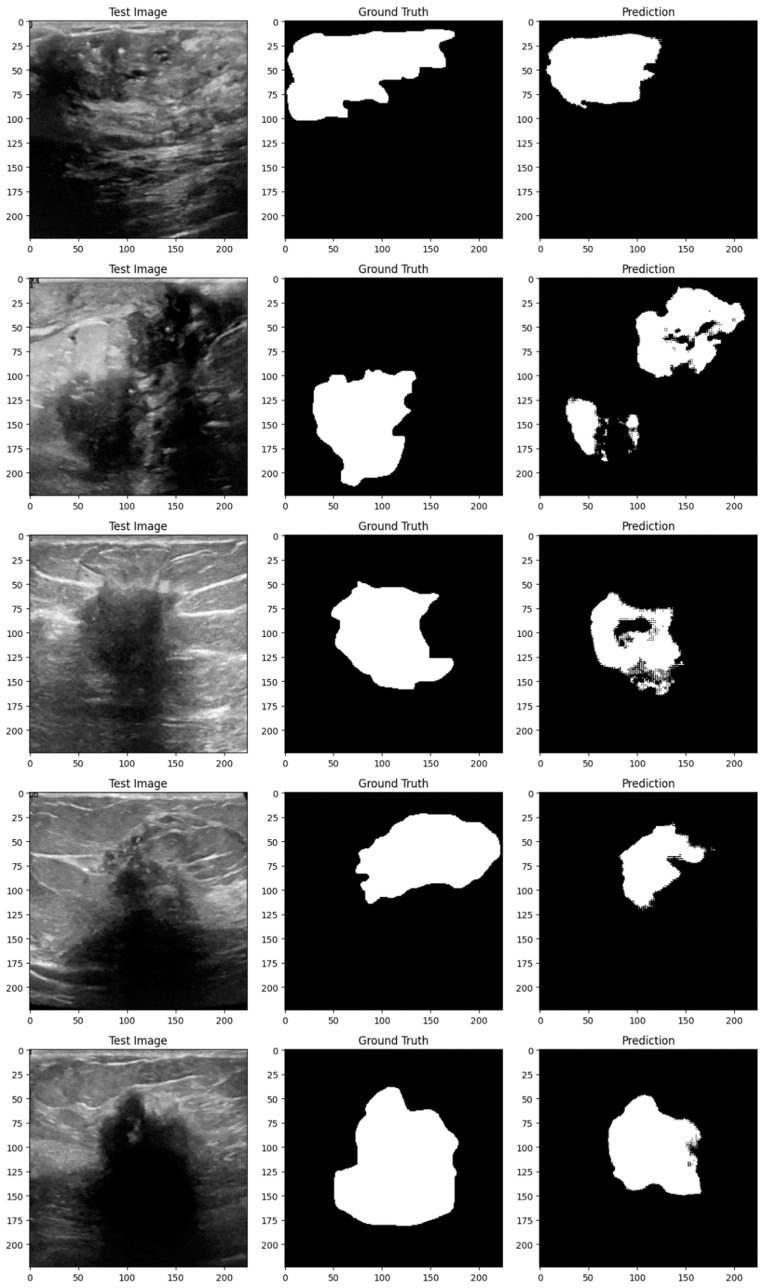
Displaying the UNet model’s predictions on test samples.

**Figure 17:**
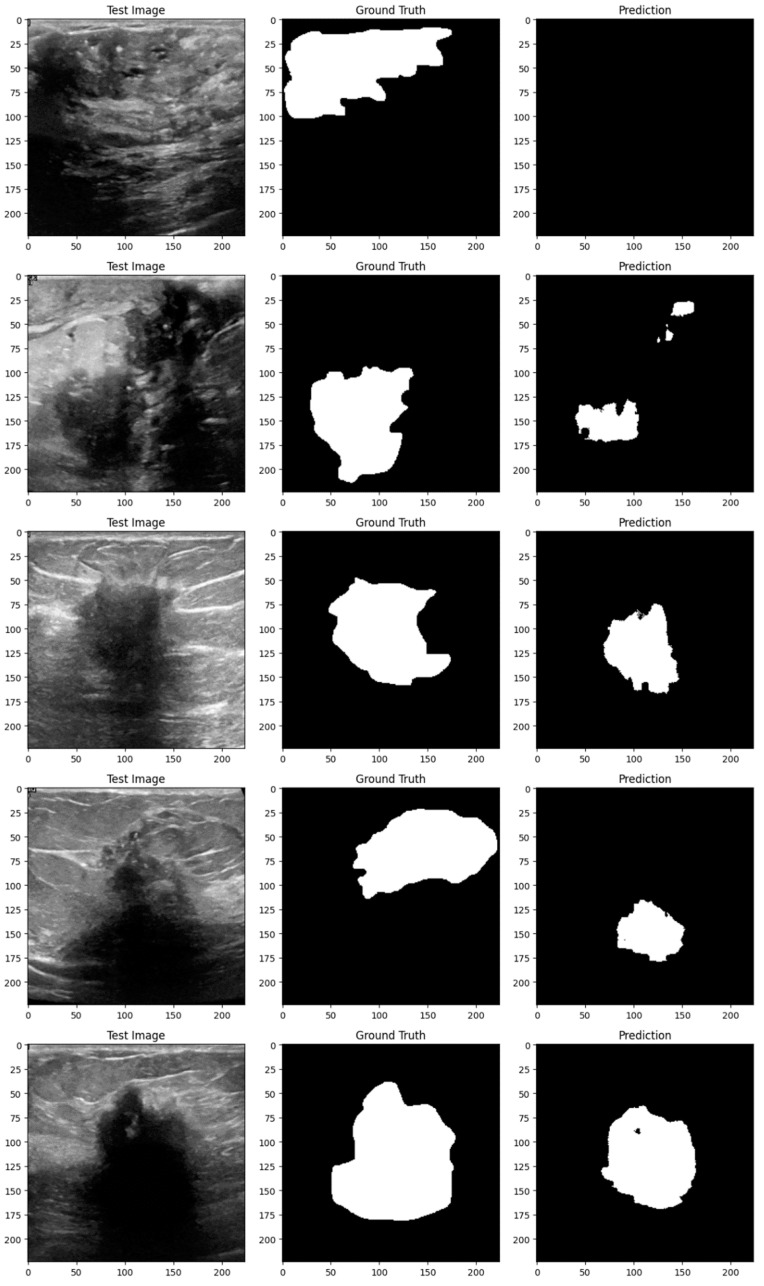
Displaying the UNet + Augmentation model’s predictions on test samples.

**Figure 18:**
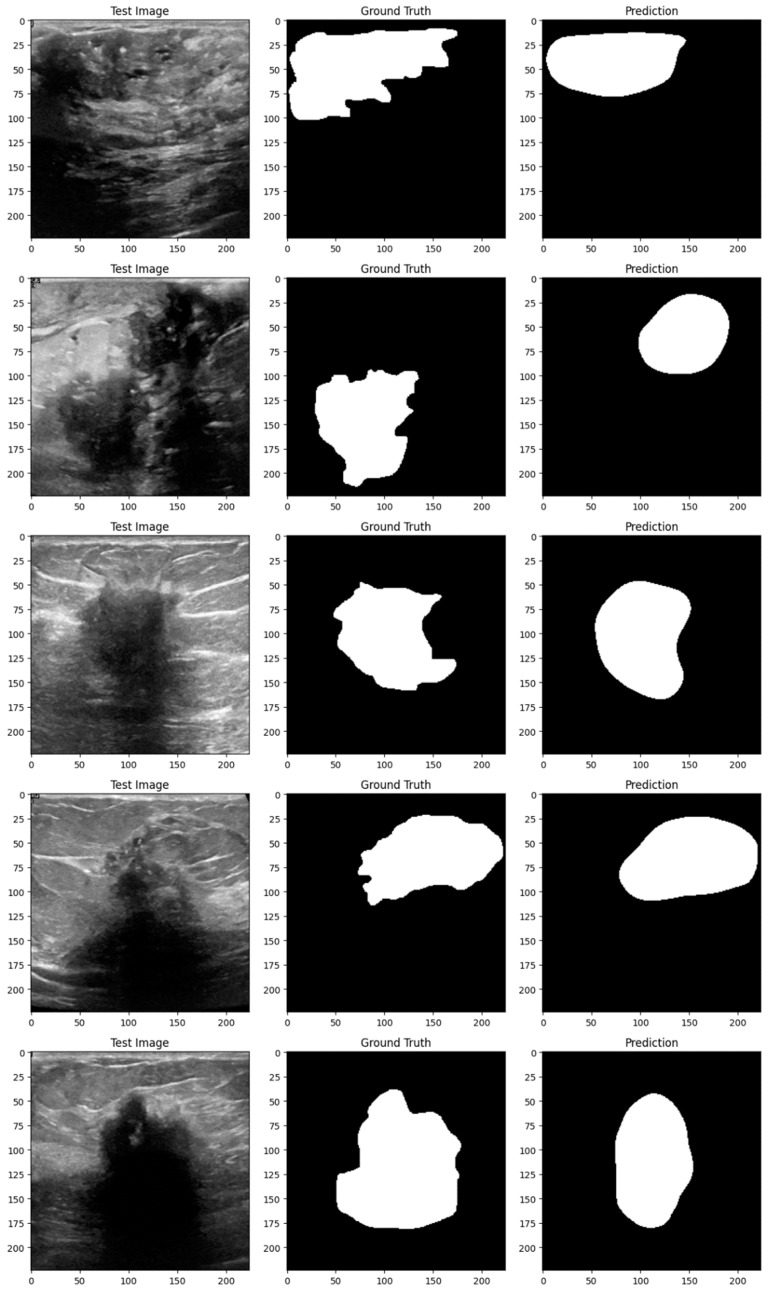
Displaying the EfficientNet + UNet model’s predictions on test samples.

**Figure 19:**
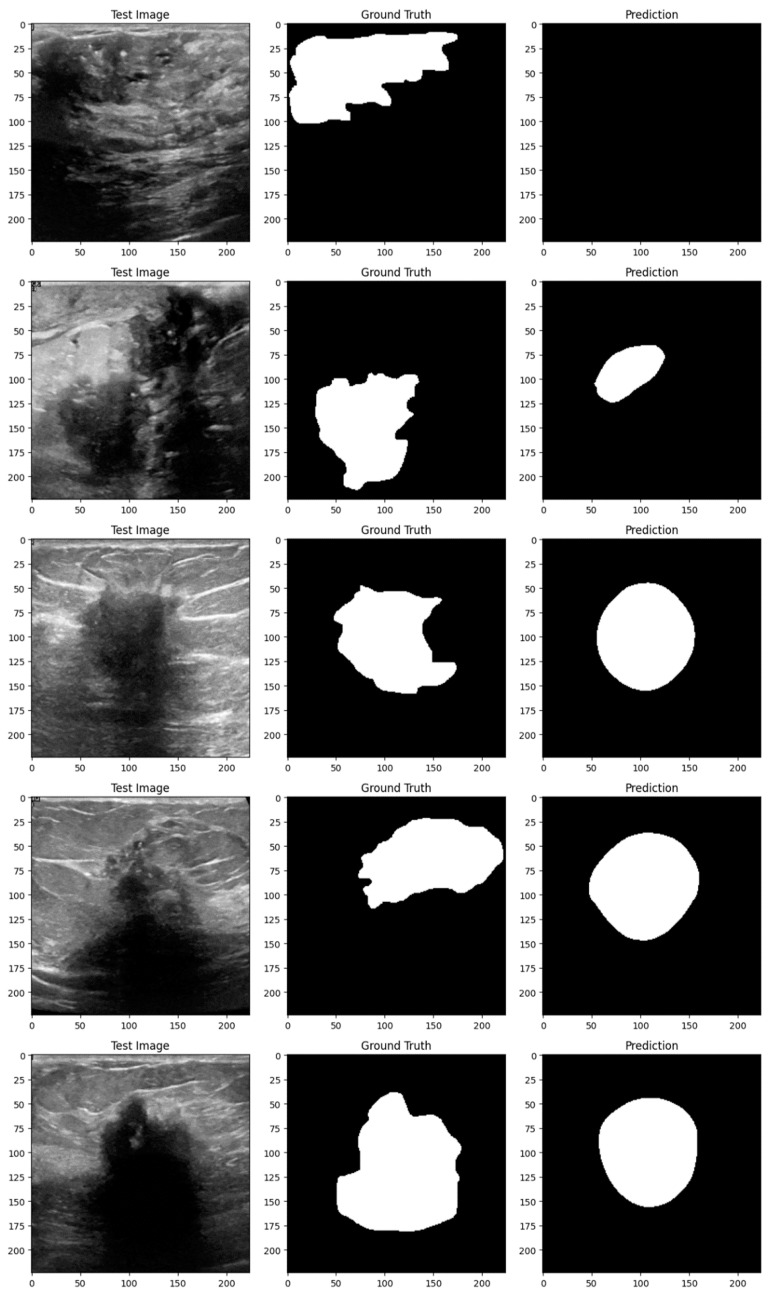
Displaying the EfficientNet + UNet + Augmentation model’s predictions on test samples.

Subsequent investigation suggests that the noise and distortions caused by the improper augmentation techniques may have confused the model and resulted in irregular learning patterns. This draws attention to a crucial factor in the training of models: augmentation techniques must improve the dataset without sacrificing its underlying organization and informational value. The instability of the enhanced model may be related to over-augmentation or the introduction of fictitious variations that do not accurately represent the variety of breast tumor images. Figure 14, Figure 15, Figure 16, Figure 17, Figure 18 and Figure 19 demonstrate the results of applying different models on test samples. 

Conclusively, the EfficientUNetViT model exhibited robust performance in segmenting breast tumors, achieving high precision, recall, and overall accuracy without the need for augmentation. Nevertheless, the use of data augmentation in this scenario resulted in reduced performance, emphasizing the importance of meticulously devising augmentation strategies that are in line with the attributes of the medical images and the particular demands of the segmentation task. This study highlights the possibility of integrating ViT with UNet topologies for medical image segmentation. It also emphasizes the significance of employing suitable augmentation strategies to further improve the model’s resilience and performance.

## 4. Conclusions

This paper presents the development and evaluation of a sophisticated breast tumor segmentation model called EfficientUNetViT, which combines ViT with a UNet architecture. The findings of our study clearly indicate that the suggested model is highly effective, as it achieves excellent levels of F1 score, precision, IoU, recall, and sice coefficient when tested on a comprehensive dataset. The ViT + UNet model, when used without augmentation, demonstrated better outcomes, highlighting its potential for precise and dependable segmentation in medical imaging. The results confirm the efficacy of combining the characteristics of ViT for collecting overall context and UNet for precise localization, resulting in a strong solution for segmenting breast tumors. 

Nevertheless, the utilization of data augmentation strategies in this investigation unveiled significant observations. Although augmentation is typically anticipated to improve the generalization of the model, our particular augmentation techniques resulted in a significant decrease in performance. This implies that the model had difficulty learning from the alterations generated by the augmentation, maybe because of the presence of noise or transformations that did not match well with the inherent characteristics of the breast tumor images. These findings emphasize the significance of meticulous design and selection of augmentation techniques in medical imaging tasks. In summary, this study highlights the potential of combining different sophisticated models in the field of medical image segmentation, while also identifying areas that require further investigation and enhancement.

There are various potential areas for further research to improve the performance and application of the ViT + UNet model for breast tumor segmentation. One approach is to use more sophisticated data augmentation methods, such as synthetic data generation or domain-specific transformations, to enhance the model’s generalizability and accuracy. Incorporating multi-modal data, such as integrating ultrasound images with other imaging modalities like mammography or MRI, can enhance tumor identification by providing more comprehensive contextual information. One possible way to enhance performance on smaller datasets like BUSI is by employing transfer learning from pretrained models on larger medical imaging datasets. Ultimately, the computational complexity of these models could be decreased by investigating more efficient transformer topologies or hybrid models. This would enhance their suitability for real-time clinical applications.

## Figures and Tables

**Table 1:** Comparative performance metrics of different models on the BUSI dataset. The table highlights the greatest value utilizing bold formatting.

Models	Precision	Recall	F1	IoU	Dice
UNet	**0.8281**	0.6122	0.6622	0.5311	0.6622
UNet + Augmentation	0.5123	0.1420	0.2087	0.1372	0.2087
EfficientNet + UNet	0.7691	0.6638	0.6916	0.5514	0.6916
EfficientNet + UNet + Augmentation	0.5978	0.3141	0.3869	0.2760	0.3869
ViT + UNet	0.7901	**0.7882**	**0.7584**	**0.6292**	**0.7584**
ViT + UNet + Augmentation	0.5200	0.2995	0.3527	0.2510	0.3527

## Data Availability

Dataset available at: https://scholar.cu.edu.eg/?q=afahmy/pages/dataset (accessed on 4 September 2024).
